# The Intersection of Ultra-Processed Foods, Neuropsychiatric Disorders, and Neurolaw: Implications for Criminal Justice

**DOI:** 10.3390/neurosci5030028

**Published:** 2024-09-23

**Authors:** Susan L. Prescott, Kathleen F. Holton, Christopher A. Lowry, Jeffrey J. Nicholson, Alan C. Logan

**Affiliations:** 1 Nova Institute for Health, Baltimore, MD 21231, USA; susan.prescott@uwa.edu.au; 2 School of Medicine, University of Western Australia, Perth, WA 6009, Australia; 3 Department of Family and Community Medicine, University of Maryland School of Medicine, Baltimore, MD 21201, USA; 4 Departments of Health Studies and Neuroscience, American University, Washington, DC 20016, USA; holton@american.edu; 5 Department of Integrative Physiology, Department of Psychology and Neuroscience, Center for Neuroscience, and Center for Microbial Exploration, University of Colorado Boulder, Boulder, CO 80309, USA; christopher.lowry@colorado.edu; 6 Law and Government, Humber College Institute of Technology & Advanced Learning, Toronto, ON M9W 5L7, Canada; jeffrey.nicholson@humber.ca

**Keywords:** neurolaw, ultra-processed foods, aggression, behavior, mental health, microbiome, auto-brewery syndrome, brain fog, monosodium glutamate

## Abstract

Over the last decade there has been increasing interest in the links between the consumption of ultra-processed foods and various neuropsychiatric disorders, aggression, and antisocial behavior. Neurolaw is an interdisciplinary field that seeks to translate the rapid and voluminous advances in brain science into legal decisions and policy. An enhanced understanding of biophysiological mechanisms by which ultra-processed foods influence brain and behavior allows for a historical reexamination of one of forensic neuropsychiatry’s most famous cases—*The People v. White* and its associated ‘Twinkie Defense’. Here in this Viewpoint article, we pair original court transcripts with emergent research in neurolaw, including nutritional neuroscience, microbiome sciences (legalome), pre-clinical mechanistic research, and clinical intervention trials. Advances in neuroscience, and related fields such as the microbiome, are challenging basic assumptions in the criminal justice system, including notions of universal free will. Recent dismissals of criminal charges related to auto-brewery syndrome demonstrate that courts are open to advances at the intersection of neuromicrobiology and nutritional neuroscience, including those that relate to criminal intent and diminished capacity. As such, it is our contention that experts in the neurosciences will play an increasing role in shaping research that underpins 21st-century courtroom discourse, policy, and decision-making.

## 1. Introduction

“*The notoriety of the Twinkie defense may not soon be rivalled…but in a system of justice based on responsibility and intentionality, the [neuropsychiatric] discoveries raise troubling questions about the morality of punishing those who may lack awareness that they are sick.*”

Kirk Makin, Legal Affairs Journalist, 1988 [1]

Neuroscientists and neuropsychologists are often called upon to provide expert testimony, especially as courts warm to scientific advances within the brain sciences [2,3]. Experts in the field assist the courts in making determinations of competency and capacity [4], including a defendant’s decision-making capacity in relation to criminal culpability [5]. In the United States, the rate of traumatic brain injury among prisoners is five times higher than the general population [6], while a recent European study using brain imaging showed that half of inmates displayed signs of brain pathology compared to 8% among healthy non-criminal controls [7]. The odds of criminal justice involvement among veterans with posttraumatic stress disorder (PTSD) are 61% higher than those without PTSD, and the odds of arrest for violent offenses are 59% higher [8]. Evidence shows that neuropsychiatric conditions are often criminalized [9]. The interdisciplinary sector that attempts to translate the rapid and voluminous advances in brain science into legal decisions and policy is known as neurolaw [10]. Neurolaw encompasses neurology, neuropsychiatry, neuroimaging, neurogenetics, neuro-microbiology, forensic neuropsychology, and related disciplines [11,12].

In the context of a criminal justice system that is based on universal assumptions related to free will and criminal intent, advances in neuroscience have profound implications for individuals, communities, and society writ-large [13,14]. For example, developmental neuroscience has upended assumptions related to sharp delineations between the adolescent and adult brain, and these findings have led to changes in the ways in which teenagers are treated by the courts [15]. A growing awareness of emerging adulthood as a distinct phase of development may have future implications. A part of ongoing discussions within neurolaw is the extent to which emerging findings require a greater responsiveness by the courts, including acceptance that criminal responsibility and blameworthiness do not sit on an even plane [16]. In addition, findings in the realm of forensic neurosciences can aid in understanding offender risk and best practices for rehabilitation [17]. However, advances in neuroscience and other biomedical sciences are often siloed away from the criminal justice field, and the relevancy of findings to the justice system is often underappreciated or relegated in importance [18,19]. 

Although the term neurolaw emerged in the early 1990s, tethered to personal injury lawsuits and the application of validated neurocognitive testing [20,21], the use of objective tests (e.g., electroencephalogram (EEG)) to limit criminal liability dates back to the 1930s [11,22]. Although relatively rare, defense teams have won acquittals (or lesser or abandonment of charges) when using neuroimaging and related assessments as part of the defense strategy [23]. Among the exciting advances in neurosciences as they relate to antisocial, aggressive, and violent behavior, is the realm of nutritional neuroscience [24,25,26,27] and its intersection with the microbiome [28,29]. In the context of human cognition and behavior, emerging research demonstrates a bidirectional relationship between dietary patterns and the microbiome [27]. That is, dietary choices influence microbiota, which, via the bidirectional microbiome–gut–brain axis, influence the brain. At the same time, microbiota appear to influence nutritional status, and perhaps even dietary-related behaviors. Neuroscience, as informed by emergent microbiome findings, provides an important mechanistic understanding of epidemiological links between diet and behavior. 

Here in this viewpoint article, we provide a retrospective analysis of one of the most famous cases in forensic neuropsychiatry, *The People v. White*, and its resultant ‘Twinkie Defense’. We use original court testimony and draw from emergent research in the fields of nutritional neuroscience [26] and the microbiome in forensics (i.e., the legalome) [18]. It is our contention that the synthesis of cutting-edge research from the top-down (nutritional epidemiology), bottom-up (mechanistic pre-clinical studies and microbiome sciences), and head-on (human intervention studies) perspectives, provides an entirely new outlook on the role of nutrition and the microbiome in forensic neurosciences. Moreover, the emergent research on ultra-processed food addiction has raised important legal questions (including individual diminished capacity and corporate culpability) under the umbrella term of food crime [30,31]. Although discussions of the Twinkie Defense are often ahistorical, we consider the broad contextual history to be an important part of its relationship to contemporary findings. That is, the Twinkie Defense did not emerge from a vacuum. Rather, it emerged from the forensic neuroscience ‘battlegrounds’ that marked the beginning of the end of Freudian pseudoscience [32,33] and untestable, subjective psychoanalytic dogma that plagued the courts [34]. 

Drawing from the PsycINFO, Google Scholar, and PubMed databases, we revisit the defense of diminished capacity through the lens of contemporary scientific research. We supplement the material found in academic databases with relevant articles drawn from media databases, including *NewsBank* and Ancestry’s Newspapers.com. This history and emergent evidence are pertinent because, as discussed below, a variation of the Twinkie Defense has recently proved successful in driving while intoxicated (DWI) cases in the United States and Europe [18]. 

## 2. The Twinkie Defense

On 27 November 1978, Daniel J. White, a disgruntled former city of San Francisco employee, used a handgun to fatally injure the mayor of San Francisco, George Moscone, and city supervisor, Harvey Milk. Shortly after the incident, White surrendered to local law enforcement. Despite his surrender and confession, White’s case went to trial with a defense of diminished capacity—an impairment not amounting to insanity, but one capable of removing the “malice aforethought” and other pre-commission mental states essential to murder. In California law, the focus was on “reduced mental capacity, whether caused by mental illness, mental defect, intoxication, or any other” [35]. White was charged with murder and a potential death penalty awaited him at sentencing. However, in White’s case, the diminished capacity defense was successful, as the jury found him guilty of voluntary manslaughter. 

The basis of White’s defense was that he was suffering from depression wherein an unhealthy diet was not merely an association, it was a contributor to his altered mental state. At trial, White’s unhealthy dietary pattern was discussed multiple times, and as we will discuss below, it was presented as a factor of causation in his cognition and behavior. For the media reporting on the trial in real time, White’s claimed dietary pattern was encapsulated in the mention, by both the prosecution and the defense, of a specific highly processed food-like product—Twinkies. Perhaps because of the product’s broad significance in American culture, Twinkies became representative of the larger aspects of an ultra-processed dietary pattern, and the defense strategy was dubbed ‘The Twinkie Defense’.

Many contemporary articles repeat the notion that the defense team barely mentioned junk food as part of the defense strategy, and that insofar as junk food was presented as a causative factor in diminished capacity, the Twinkie Defense is claimed to be a “myth” [36,37]. Even textbooks in the category of forensics claim that White’s diminished capacity defense was exclusively about depression and the verdict had nothing to do with Twinkies or any such ultra-processed foods [38,39]. In a multi-author academic text on testimony from brain science experts, it is claimed that “The trial for double-murder concluded without reference to White’s poor eating habits. It was not until after the jury’s verdict and the judge’s sentencing were made public that the issue of sugar consumption as an influence on White’s criminal behavior was raised” [40]. One of the most highly regarded books in biological criminology repeats this same false claim that Twinkies “were never actually brought up at Dan White’s trial” [41]. Retrospective articles in high profile news outlets also claim that a mere “throwaway remark” about junk food by the defense, led to an overblown media sensation and the development of a “myth” [36]. However, our analysis of the actual trial transcripts [42], as below, show that such claims of “myth” do not hold up under scrutiny. Taken as a whole, the recorded testimony and closing arguments demonstrate that the words candy, cola, Twinkies, cupcakes, potato chips, junk food, sugar, or related variants such as brand name soft drinks, were used over two dozen times. Trial transcripts quoted below show that defense attorney Douglas Schmidt leads the defense expert witness, Dr. Martin Blinder, *beyond* association and into causation:

**Defense Attorney**: Doctor, you have mentioned this ingestion of sugar and sweets and that sort of thing…does that have any significance, or could it possibly have any significance?

**Dr. Blinder**: Well, I think, Mr. Schmidt, there are probably three factors that are significant. First, there is a substantial body of evidence that in susceptible individuals large quantities of what we call junk food, high sugar content food with lots of preservatives, can precipitate anti-social and even violent behavior. There have been some studies, for example, where they have taken so-called career criminals and taken them off all their junk food and put them on milk and meat and potatoes, and their criminal records immediately evaporate. There have been a lot of studies in which individuals who are susceptible to these noxious stimuli, when given these noxious stimuli will undergo complete change and engage in behavior which they normally would not. That’s number one.

The crucial part in relation to the Twinkie defense is that Blinder places the ingestion of junk food, which he had just referred to as “noxious stimuli”, into the causative mix: 

**Dr. Blinder**: If it were not for all the tremendous pressures on him the weeks prior to the shooting, and perhaps if it were not for the ingestion of this aggravating factor, this junk food, with all three factors, did not impinge upon him at the same time, I would suspect that these homicides would not have taken place.

Obviously, Dr. Blinder referred to junk food ingestion as the aggravating factor. His reference to a “substantial” body of evidence demonstrating behavioral changes in association with junk food consumption—acting in ways and doing things that are out of the ordinary—provides an inference of a scientific discipline, a specialty. At the time, there was a peppering of historical (low-quality) research indicating that hypoglycemia induced by unhealthy dietary choices could influence EEG results and other neurolaw-related markers [11,43]. Critically, Dr. Blinder underscores that it had been shown that abnormal behavior, even for career criminals, can be remediated by a return to what a reasonable person would assume to be a diet of minimally processed foods. Thus, we have an expert who is informing the jury that science has shown that a diet is to aggression, what smoking is to cancer—an aggravating factor of potential causation. 

It is likely that the jury absorbed that messaging because courtroom media picked up on it in real time; following Blinder’s testimony and before jury deliberations had even begun, the *Chicago Tribune* reported in a nationally-syndicated article that the defense “has been so short on explanations [for diminished capacity] that it seems to have chosen, as one possible rationale, White’s junk food diet”, and the reporting added that the defense psychiatrist was convinced that White’s “deep depressions were escalated by the sugar-heavy diet” [44]. The key word is *escalated*, which clearly indicates that independent observers were interpreting elements of aggravation and causation. After hearing Dr. Blinder’s testimony, journalist Paul Krassner jotted down the words “Twinkie Defense” and placed them in his real-time reporting for the *San Fransisco Bay Guardian* [45]. 

There is additional evidence that White’s claimed junk food diet was being broadly interpreted as a causative factor—the reaction by the prosecution. In Assistant District Attorney (ADA) Thomas Norman’s rebuttal, the prosecution brought in Dr. Roland Levy as their own expert witness. Levy was led into questioning designed to refute the causal diet–crime connections: 

**ADA Norman**: Doctor Levy, are you familiar with any studies and any prevailing scientific bodies of thought relating to the ingestion of sugar, foods with preservatives such as what’s commonly known as junk foods and including, for example, chocolate cupcakes of Twinkie variety, Coca-Cola, candy bars and potato chips, for example, as those relate to being causative factors in influencing anti-social or sociopathic behavior?

**Dr. Levy**: I am unaware of any prevailing psychiatric opinion that such factors are significant in relationship to any type of mental illness. And I am unaware of any publications in major journals which state that. 

Dr. Levy is then cross-examined by White’s defense, chipping at his credibility to discuss nutrition and violence.

**Defense**: Doctor, I think you testified this morning that you were unaware of any prevailing attitudes or opinions in psychiatry with regard to the ingestion of sugar, or that type of food, is that fair?

**Dr. Levy**: Yes.

**Defense**: Would you agree there has been research in that area?

**Dr. Levy**: There has been.

**Defense**: There has been much research and publications in that area?

**Dr. Levy**: Publications, yes.

**Defense**: And, of course, you have no training or experience with regard to that particular facet of the field?

**Dr. Levy**: I have never gone into that in any extent at all. 

These exchanges clearly demonstrate that the prosecution was well aware of the defense tactics of foods as “causative factors”, and the defense was undermining the expertise of the sole prosecution witness. The problem with Dr. Levy’s response, at least for the prosecution, is that it left the reasonable person with the idea that although connections between highly processed food and violence may not be part of “prevailing” and “major” psychiatric thinking, it could still be part of lesser-known science. 

Defense attorney Schmidt opened the door to this novel research in his closing arguments. Schmidt made it clear that even though he could not say definitively that high sugar foods precipitate violence, and that the defense psychiatrist “*didn’t go on and on about ‘eat a Twinkie, and go crazy’*”, the defense expert had provided important testimony that supports the idea of junk food as an aggravating factor [46]. In other words, one Twinkie was not the culprit, it was a pattern of consumption that intersects with and aggravates mental health vulnerabilities. In his closing arguments, Schmidt revealed his own intentions as to why he called Dr. Blinder: “*I don’t know whether there is a relationship between sugar and violent behavior. I don’t know. A lot of people think so. It’s hypoglycemia. If there is some possible relevance regarding that then I want somebody to come in and tell me what it is. And that’s why I called Dr. Blinder*”. Schmidt openly acknowledges that Dr. Blinder was brought in to discuss the science of food and antisocial behavior. Schmidt tells the jury that the expert witness for the prosecution, Dr. Levy, essentially called the theory “nonsense” because he was ignorant of the science, “*and if I* [referring to Dr. Levy with sarcasm] *don’t know about it then it’s nonsense*”. Schmidt then goes further with the inferences and refers to science outside mainstream dogma: “*Whether or not ingestion of foodstuff with preservatives and sugar in high content causes you to alter your personality somehow, or causes you to act in an aggressive manner, I don’t know…there is a minority opinion in psychiatric fields that there is some connection and there very well may be*. *And if there is a connection, we are working on a reasonable doubt system here. If you have some reason to doubt something, it’s got to be brought up to you because that’s a possible reason for doubting that this was a cold-blooded premeditated murder*” [47].

## 3. Neuroscientist and Jury Reactions

Public reaction to the Twinkie Defense and the trial outcome was not one of support. In the immediate aftermath, California and other states moved swiftly to reduce or eliminate opportunities for diminished capacity as a defense. An article in the *San Franscico Examiner* noted that “the cream-filled rolls [Twinkies] came to symbolize the public’s disenchantment” with neuropsychiatric testimony [48]. 

Commenting on the trial in the American Bar Association’s journal *Litigation*, neurologist Dr. Harold L. Klawans concluded that the defense presentation of White as someone with depression would not have been enough—it was Dr. Blinder’s diet-causation theory that sold the jury [49]. *Newsweek* magazine’s conclusion was that Dr. Blinder essentially informed the jury “*that White’s compulsive diet of candy bars, cupcakes, and Cokes was evidence of a deep depression-and a source of excessive sugar that had aggravated a chemical imbalance in his brain*” [50].

While detailed jury-derived explanations for the verdict are lacking, at least one juror stated, “the psychiatric testimony was highly influential. It was mainly what we based the verdict on… it sounded like Dan White had hypoglycemia”, another said, “some people believed it [junk food as a key factor]”, and another added, “I don’t know that much about it [food and behavior]. It’s new research” [51]. From these limited public statements, we know that at least one juror was listening to the defense’s pitch that hypoglycemia can make humans behave abnormally; some on the jury believed the defense propositions, and the latter juror absorbed Blinder’s pitch that the topic was, if nothing else, an emerging science. 

In sum, the Twinkie Defense is not a “myth”. There is ample evidence that the defense presented the jury with a scenario in which the defendant’s mental state was aggravated by a reliance upon junk foods, and that aggravation, in the words of the defense attorney in closing arguments, caused the pot “to boil over”. There is also clear evidence that the presentation of the defense did not occur in a vacuum: in the late 1970s in San Francisco, the idea that nutrition could influence behavior was within the cultural petri dish. If there is anything mythical about the Twinkie defense, it is the notion that Twinkies, per se, were presented as a singular item of causation. They were not. The food-like product was simply a culturally relevant symbol of what are now referred to, in the scientific literature, as ultra-processed foods. While we have taken the opportunity in the current essay to provide corrective history, our larger aim is to consider whether or not, four decades later, advances in science lend credibility to the essence of the defense. 

## 4. From Pseudoscience to Contemporary Science

In his critical article in *Litigation*, neurologist Klawans argued that the prosecution in the White case should have eviscerated the idea that highly processed foods were an aggravating factor: “*the Twinkie defense would have been easy to demolish. Mere child’s play. It is not the product of science… There are no data. There never have been. What passes for data are anecdotes and apocrypha, not controlled scientific studies*” [49]. While that may have been true in 1979, emergent research from various branches of science supports the idea that dietary patterns, and even the more short-term consumption of macronutrients and dietary additives, can influence brain and behavior [27]. As discussed below, bottom-up research (e.g., microbiome and preclinical) combined with top-down (e.g., nutritional epidemiology) research has been amplified by a collection of recent intervention studies showing that diet is important in behavioral outcomes. Emergent research includes an essential ingredient that was missing from the Twinkie Defense—plausible and reproducible biophysiological mechanisms providing explanations for links between diet and aggression. This includes brain imaging studies showing that the dietary manipulation of gut microbes has the potential to alter the activity of brain regions that control the central processing of emotion and sensation [52]. 

Before discussing critical findings in the realm, it is worth pointing out that the development of the academic NOVA Food Classification system [53], with its categorical delineation of ultra-processed foods, has allowed international researchers to make a firm case that such foods are detrimental to health, including mental health [54,55]. Ultra-processed items are foods and beverages that typically contain high amounts of refined sugar and/or fats and/or sodium, contain low amounts of naturally occurring dietary fiber and phytochemicals (as found within the minimally processed food matrix), and are typically inclusive of additives such as emulsifiers, flavor enhancers, colors, and isolated fiber and antioxidants that have been removed from their original food matrix; commercially available foods that contain five or more ingredients are generally in the ultra-processed category [56]. Compared to minimally processed foods, ultra-processed foods are also notable for what they typically do not contain—polyphenols and live, non-pathogenic microbes with potential benefits to mental health [57,58]. Dietary patterns high in ultra-processed foods (and/or high fat/sugar) have recently been linked to mental distress [59], various mental disorders [60,61,62,63,64,65,66,67], impulsivity [68,69], diminished concern for future consequences [70], and antisocial/aggressive behavior [71,72,73,74,75,76,77]. Studies using neuroimaging have shown that unhealthy dietary patterns are associated with smaller hippocampal and brain volumes [78,79]. Remarkably, there are indications from human research that even short-term (four day) diets high in added sugars and saturated fat can have a detrimental influence on hippocampal-dependent learning and memory [80]. 

Despite the large scale of many of the epidemiological studies, they do not provide evidence of causation. However, at least one recent study with a tightly-controlled design (matching macronutrients and total calories in an institutional setting) has shown that ultra-processed foods differ from their minimally-processed counterparts in that they encourage greater caloric consumption and weight gain [81], and produce differences in the human metabolome [82]. In a throwback to *White*, researchers are demonstrating that vulnerable individuals have the potential to become addicted to ultra-processed foods [83,84]. In the context of mechanisms described below, ultra-processed food consumption has recently been associated with low-grade systemic inflammation [85,86,87] and appears to influence systemic metabolites and energy uptake via disturbances to the gut microbiome [88,89,90,91]. In recent years, scientists have also developed a more sophisticated understanding of the ways in which specific nutrients, ranging from vitamins and minerals to omega-3 fatty acids, influence the structure and function of the brain [92]. Moreover, dietary fiber and naturally occurring phytochemicals (e.g., polyphenols and related chemicals that give food their colors, taste, and texture), constituents of minimally-processed foods, are also involved in cognition and behavior via direct and indirect effects on nervous system health [93]. 

## 5. Mechanistic Pathways

There are a number of ways in which dietary patterns and individual dietary components can influence (i.e., promote the integrity of, or compromise) brain structure and function. Either directly, or through metabolic reactions, multiple nutrients (and non-nutritive phytochemicals such as polyphenols) play critical roles in maintaining the integrity of neuronal membranes, neurotransmitter production, neurogenesis, and protection through antioxidant defense systems [94,95]. Ultra-processed foods and the synthetic additives within can compromise dopamine and serotonin neurotransmission, through influence on gene transcription and, as discussed below, inflammation [96,97]. Preclinical studies show that these foods and the additives within can disrupt the amygdala–hippocampal complex, which, extrapolated to humans, suggests an influence on the frontolimbic emotion regulation brain network; indeed, ultra-processed food consumption in humans has been associated with lower volumes in mesocorticolimbic areas of the brain [98]. 

In the context of healthy dietary patterns and the brain, there is significant nutritional complexity in structure–function connections; that is, single nutrients often work in relation to others. For example, the omega-3 fatty acids docosahexaenoic acid (DHA) and eicosapentaenoic acid (EPA) play important roles in neuronal structure and signaling; however, these fatty acids are dependent on B vitamins for transport [99]. Another example is magnesium, which has been linked to antisocial behavior [100]. It is difficult to pinpoint precise mechanisms because magnesium plays a role in hundreds of enzymatic reactions and interacts along with other nutrients. Pertinent to our discussions of dietary glutamate below, low levels of magnesium exacerbate neuronal excitotoxity [101], while omega-3 fatty acids protect against monosodium glutamate neurotoxicity [102]. There is also evidence that chronic psychological stress increases biophysiological demand for a variety of micronutrients [103]. This might be especially the case with nutrients involved in the antioxidant defense system and those involved in immune function, including zinc, magnesium, iron, and various B vitamins [103]. 

One mechanistic area that has received considerable attention is the role of dietary patterns and individual dietary components on neuroinflammation. Volumes of research now point to inflammation as a causative factor in neuropsychiatric conditions. Included in this research are studies linking elevations in immune proteins (e.g., cytokines) in adults with aggressive behavior and/or aggressive tendencies [104,105,106,107]. Both pre-clinical and human research indicates that chronic microglia activation in the brain may contribute to various neuropsychiatric disorders, a loss of impulse control, addiction, and aggressive behavior [108,109,110]. Neuroinflammation is related to excitotoxity (the overactivity of neurons) and oxidative stress within the central nervous system. Together, neuroinflammation, oxidative stress, and excitotoxicity, are known as the ‘neurotoxic triad’ and each component can influence the other in a potentially self-sustaining and synergistic manner [111]. Conditions for the neurotoxic triad are made worse by psychological trauma and acute and chronic stress which compromise the structure and function of the normal blood–brain barrier (BBB). This allows the infiltration of chemicals normally excluded to the periphery, including inflammatory cytokines, to gain central access [112]. Diet plays a crucial role in the neurotoxic triad because certain dietary patterns, such as those rich in vitamins, minerals, fiber, omega-3 fatty acids, and phytochemicals, have been shown to limit neuroinflammation [111]. On the other hand, patterns rich in high-sugar/high-fat ultra-processed foods, often containing various emulsifiers and flavor enhancers (e.g., free-form glutamate, monosodium glutamate (MSG), and aspartame), have been linked with the neurotoxic triad [113].

Animal research using germ-free models indicates that stress can directly elevate systemic inflammation in the absence of microbiota [114]. However, a growing body of international research demonstrates that the gut microbiome plays an important mediating role in the links between dietary patterns (and specific components of diet) and neuroinflammation [115]. As discussed in the next section, emerging research suggests that the dysbiosis (disturbances to the microbiome) observed in most neuropsychiatric conditions is not merely an association or consequence of a disease or disorder [116].

## 6. Microbiome and the Legalome

Although much of the human research linking microbial alterations with emotional regulation and neuropsychiatric disorders is cross-sectional [117], researchers are moving beyond mere associations [118]. Dietary patterns high in ultra-processed foods (vs. minimally processed, polyphenol, and fiber-rich diets) have been linked to gut dysbiosis [91,119,120,121]. Individual components of ultra-processed foods, such as synthetic emulsifiers and dietary excitotoxins, have been linked to dysbiosis [122,123]. On the other hand, human research shows that targeting the microbiome via diet (e.g., whole grains, onions, leeks, cabbage, oats, and fermented foods) can reduce psychological stress and improve mood [124,125]. 

The ultra-processed food induction of gut dysbiosis is important because preclinical studies are indicating that gut microbes play a causative role in cognitive–behavioral disturbances, including aggression and antisocial behavior. For example, when the fecal material of animals with diet-induced dysbiosis is transplanted into otherwise healthy animals, these recipients have observable behavioral disturbances as found in the dysbiotic donors [126,127,128]. These behavioral signals via microbiota transfers have also been observed when the fecal material originated from human donors with behavioral disorders [129,130,131,132]. When animals are the recipients of fecal material obtained from human donors with mild cognitive impairment, recipients experience learning and memory problems in association with impaired cerebral glucose uptake [133]. Of relevance to addiction, microbiota from alcohol-dependent patients induced the behavioral alterations associated with alcohol dependence in recipient lab animals, including increased anxiety- and depression-like behaviors, reduced exploratory and recognition memory, and higher alcohol preference; these behavioral changes were accompanied by objective brain-related signals known to be associated with alcohol dependence [134]. Remarkably, the transfer of fecal material from human infants with microbial disruptions (from the administration of antibiotics) leads to aggressive-like behavior in recipient lab animals, observations not seen with transfer of microbiota from healthy infants [135]. When combined with other preclinical fecal transfer studies, the emerging picture is that the human equivalents of depression, anxiety, and behavioral disorders are (at least to some degree) rooted in, and transmissible by, gut microbes [136]. 

The mechanisms explaining such observations are beginning to take shape; in particular, gut dysbiosis leads to intestinal permeability (a more porous intestinal lining, or so-called ‘leaky gut), increased levels of circulating immune chemicals, most notably proinflammatory cytokine responses, and an ongoing low-grade inflammation [137,138]. This low-grade inflammation is considered a risk factor for a variety of neuropsychiatric conditions [107,139]. In animals, exposure to ultra-processed diets can increase the permeability of both the gut and the aforementioned BBB [140]. This low-grade inflammatory cascade and associated metabolic dysregulation can influence mood and aggression [141,142]. Intestinal permeability allows gut microbial breakdown products, such as lipopolysaccharide endotoxin (LPS), to enter circulation. LPS provokes inflammatory cytokine release and enhances the potential of dietary excitotoxins such as MSG to promote neuroinflammation and the dysfunction of neurotransmission [143]. Animal studies show that MSG can influence serotonin, dopamine, and norepinephrine levels [144,145], and alter the gut microbiome [146]. The formation of bioactive polyphenolic metabolites via microbial activity in the gut lumen, with preferred delivery to the mammalian brain [147], appears to play an important mechanistic role in mental and cognitive health [93].

Since research is pointing toward a causal role for gut microbiota in systemic inflammation [148], scientists are actively pursuing gut microbial signatures that can tie together intestinal permeability, systemic low-grade inflammation, and the risk of aggression [149,150]. It is also true that alterations in the gut microbiome and the cascade of systemic low-grade inflammation can be mediated from the top-down, i.e., stress activates brain to gut pathways, altering the gut microbiome [151]; these stress-induced changes to the microbiome appear to drive and maintain psychological symptoms via microbial influences on various metabolic pathways [152]. In human research, reports point toward linkages between select microbes that appear to be involved in reactive aggression, such as *Lachnospiraceae* and *Eubacterium* genera, that thrive with unhealthy dietary patterns absent in phytochemical-rich vegetables [153]. 

**Figure 1:**
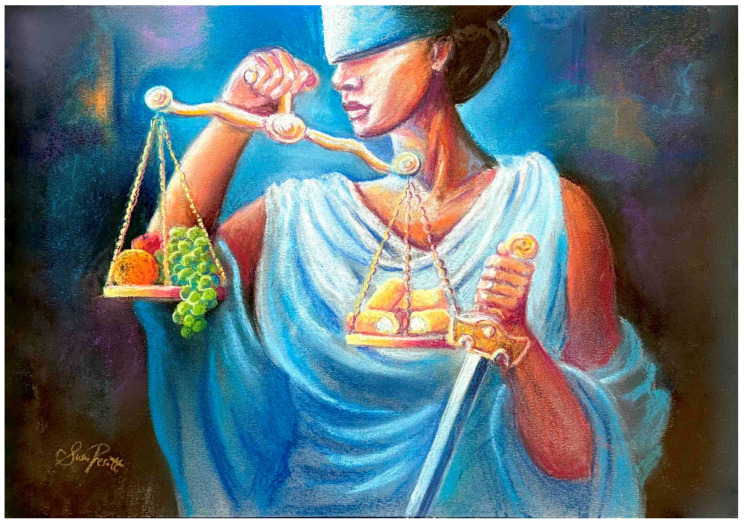
Recent cases involving the dismissal of driving while intoxicated charges after lab-proven internal alcohol production (i.e., “auto-brewery” via food and microbe interactions, without alcohol consumption) demonstrate that the courts are opening to the legalome and variants of the Twinkie Defense (used with permission of Susan L. Prescott).

The rapidly emerging research in microbiome sciences has already intersected with criminal courts and considerations of culpability. As described in detail elsewhere, cases of forensically relevant alcohol production from the human gastrointestinal tract (i.e., auto-brewery syndrome), without any consumption of alcohol-containing beverages/foods, are increasingly described in the medical literature [18]. This internal alcohol production, which can bring a person close to or above legal limits of blood alcohol, is a product of dysbiosis, most notably the overgrowth of yeasts such as *Candida* and bacterium such as *Klebsiella* [154]. Since the acute microbially driven alcohol production is via high-sugar, ultra-processed drinks/foods (with dysbiotic microbes acting on ingested sugar to produce alcohol), the resultant inebriated state and “brain fog” raises questions of diminished capacity and criminal intent. In both the United States and Europe, cases of DWI have recently been dismissed due to lab-proven internal alcohol production (i.e., with zero consumption of any alcohol containing product) [18] (Figure 1). It is important to note that blood alcohol levels far below DWI-related legal standards are associated with cognitive changes and a higher risk of injury [155]. The recent court rulings are, at least to some degree, a validation of the ideas first floated around the time of *The People v. White*, and indicate that the courts are willing to accept lab-proven connections between diet, gut microbes, and the production of metabolites that can otherwise influence cognition and behavior. As noted in a recent editorial in *Trends in Microbiology*, the available microbiota–gut–brain axis research is already forcing important policy questions upon society, including efforts to remedy systemic health inequalities [156]. We suggest that the legalome (microbiome and omics science applied in forensic and legal psychology) will be part of those salient policy discussions. 

## 7. Intervention Studies

The case for claimed causality is strengthened by emerging nutritional intervention studies. In the immediate aftermath of *The People v. White*, some researchers began examining the relationships between diet and antisocial behavior in correctional settings. In a series of quasi-experimental studies across twelve different US juvenile correctional institutions, investigators swapped out foods with high amounts of added sugar and refined fats for similar, less processed options [157,158,159,160]. Combined, the studies involved several thousand juveniles with the results showing an average 47% reduction in documented offenses, infractions, and other indicators of antisocial behavior. These included reductions in overt violence, acts of theft, verbal aggression, and insubordination to corrections personnel [161,162].

While encouraging, these dietary interventions studies from the 1980s lacked tightly controlled comparison groups and double-blind design, and undoubtedly suffered from expectancy effects among participants. With a better understanding of potential mechanisms, contemporary researchers have revisited the topic of nutritional neuropsychiatry with more tightly controlled research designs. Many of the emerging nutritional neuroscience/psychology studies examine depression as an outcome. In the context of forensic psychology, the positive influence of diet on depression is important because research has shown that depressive symptoms are associated with cognitive disturbances related to legal capacity [163,164,165,166,167,168], enhanced risk-taking [169], and deficits in social problem-solving [170]. The SMILES trial (*n* = 67) found improved ratings of depression on a clinical rating scale with a healthy diet intervention compared to a social support control group [171]. It appears that the positive results of the SMILES trial were related to the elimination of ultra-processed foods, rather than the introduction of specific healthy foods [172]. Research suggests that mental health can be influenced by relatively short-term dietary changes. For example, in a randomized dietary intervention study involving young adults (*n* = 101), a switch to a healthy dietary pattern (vs. habitual diet) lowered the symptoms of depression in three weeks; the significant improvements were also documented at a 3 month follow-up [173]. In a multi-center, randomized controlled trial (*n* = 292), researchers reported that a low-fat, low-glycemic index, plant-based diet intervention improved mental outlook and work productivity in adults [174]. These studies are joined by similarly-designed randomized controlled intervention studies, demonstrating improved mental health with adherence to dietary patterns emphasizing fruits, vegetables, lean meats, fish, and whole grains, at the exclusion of highly processed snacks and fast food [171,175,176,177,178,179].

In an older non-randomized intervention trial, the addition of citrus juice in a juvenile correctional facility was associated with a 47% reduction in antisocial behavior [180]. More recent research shows that the oral intake of juices rich in naturally occurring flavonoids improves mood in young adults with depressive symptoms [181], and lower scores on anger/hostility measures in otherwise healthy adults have also been noted [182]. In addition, human studies have also evaluated the response to the removal/limitation of components commonly found in ultra-processed foods. Removing dietary excitotoxins (e.g., aforementioned free-form glutamates, MSG, and aspartame) shows benefit along a number of neuropsychiatric lines [183,184]. The metagenomic analysis of fecal material is allowing researchers to evaluate the accuracy of self-reported food intake among research participants, advances that will undoubtedly strengthen the validity of outcomes in future studies [185]. 

Although our focus here has been on dietary patterns, rather than isolated nutrients, it is worth pointing out that some of the important constituents of healthy dietary patterns, such as omega-3 fatty acids, may play a role in curbing aggression and antisocial behavior. For example, a recent meta-analysis of 28 randomized-controlled trials (*n* = 3918) concludes that omega-3 fatty acids modestly, but significantly, reduce aggression in both children and adults [186]. The study of dietary supplements (e.g., multivitamin–mineral formulas, zinc, magnesium, vitamin D, and food-derived polyphenols) for the reduction of aggression and antisocial acts (especially in correctional settings) remains limited, although encouraging results have been noted [187,188]. In addition to the direct influence of omega-3 fatty acids and other individual nutrients on neurotransmission, and inflammation-lowering effects, beneficial outcomes may be mediated by influence on the gut microbiome [189]. Related to this are the growing number of human studies indicating that ‘psychobiotic’ agents (e.g., probiotics and prebiotics) can influence social decision-making [190], buffer stress and improve mood [191,192], and lower aggressive thoughts [193], aggressive actions [194], and impulsivity [195].

## 8. Where to Next?

In order for nutritional neuroscience to have increased application in the context of neurolaw, many questions will need to be answered, especially those that close the gap between correlation and causation. Even though there is an increasing number of controlled intervention studies showing that healthy dietary patterns (and the avoidance of ultra-processed foods) can influence mood and cognition, these have typically not considered justice-involved people. Future studies should include justice-involved populations, pairing controlled dietary interventions with behavioral outcomes and objective markers, including gut microbiome indicators, inflammatory cytokines, indicators of leaky gut, and those drawn from omics technologies (including genomics, epigenomics, metabolomics, and/or transcriptomics). Given the emerging research on the gut microbiome and temperament [196], there is a need to study the microbiome in justice-involved populations [197], while examining potential dietary links and the neurobehavioral consequences of dysbiosis [198].

The area of ultra-processed food addiction and its potential relationship to antisocial behavior is worth pursuit. Accumulating human and preclinical research show that ultra-processed foods have addictive properties, and attempts to stop consuming such products induce withdrawal-like signs and symptoms [199]. Neuroimaging studies suggest there may be common pathways underpinning the observation of reward–behavior in individuals engaging in antisocial activity [200]. Recent evidence suggests that the experience of victimization (e.g., through violence or bullying) increases the likelihood of ultra-processed food consumption [201]. Since many offenders have a prior history of being victimized, this relationship is worthy of analysis. To what extent is the chronic consumption of hyperpalatable foods an attempt at reducing stress [202]? Ultra-processed food consumption has also been linked to other known risk factors for antisocial behavior, including alcohol consumption and illicit drug use [203]. The microbiome may provide useful markers of vulnerability to ultra-processed food addiction, and guide intervention efforts [204]. Clinicians and researchers now have validated tools which can be used to screen for the symptoms of ultra-processed food addiction [205]. 

Another area worth pursuit is whether or not ultra-processed foods can influence neurocognition above and beyond any outcomes that can be traced to macronutrients such as high sugar content. Although ultra-processed foods can contain high amounts of sugar, the NOVA classification system is not ideal for separating out high-glycemic index foods [206]. Consumers can wade through foods that are technically ultra-processed and still find items that are low in sugar and inclusive of significant levels of nutrients. On the other hand, relying only on the sugar or fat content of a food or beverage can overlook the ways in which an artificial sweetener (e.g., aspartame) or flavor enhancer (e.g., MSG and its many variants) could influence neurocognitive outcomes. One way to examine this would be to study the effects of different meals (with manipulated levels of processing/additives) in laboratory designs that are intended to (potentially) lead to signs of aggression and antisocial behavior. Simulated long-distance urban driving, intended to assess fatigue and ‘road rage,’ is an example where post-prandial effects could be studied. Research already indicates that aggression may be a common response to reactive hypoglycemia [207,208]; however, it is unknown whether aggression and antisocial behavior might differ when lab-induced self-control fatigue is examined when participants first consume meals differing in processing levels, sugar content, and excitotoxin additives. 

The study of nutritional neuroscience as it intersects with the courts has the potential to illuminate some of the vague diagnostic conundrums common to clinical medicine in general, and neurologists in particular. For example, “brain fog” is a term often used by patients as a way to express a long continuum of a lack of attentional focus and cognitive fatigue [209]. Brain fog can be a descriptive for the depersonalization and derealization that is associated with some criminal acts [210,211,212]. Recently, validated instruments have emerged to assess brain fog [213]. Brain fog is common to individuals with gastrointestinal complaints, and brain fog questionnaires are capable of distinguishing between established measures of obvious cognitive impairment (e.g., the Montreal Cognitive Assessment and the Digital Symbol Substitution test) and the mental fatigue, exhaustion, drowsiness, and difficulty thinking and focusing that otherwise characterizes brain fog [214]. These brain fog assessments can be paired with objective neuroimaging, EEG testing, biological samples (omics-related), and mobile technology-assisted ‘ecological momentary assessment’ as a way to better understand how diet might intersect with cognition and behavior. How might a concept such as brain fog relate to aggression and unplanned criminal activity (so-called irresistible impulse), and what are its biological underpinnings? Since brain fog has been associated with dysbiosis and an elevated production of D-lactic acid [215,216], and elevated lactic acid, especially D-lactate, has been linked to anxiety, aggression, and signs/symptoms that mimic alcohol inebriation [217,218,219,220,221], once again, microbiome insights might be helpful. There is considerable variation in plasma D-lactate levels among the general population, and it is worth noting that the microbial species often linked to auto-brewery syndrome, *Klebsiella pneumoniae* [18], is also efficient at converting glucose into D-lactate [222]. The growth of *Klebsiella pneumoniae* is promoted by dietary patterns dominated by sugar and simple carbohydrates, with absent naturally occurring fiber [223].

In relation to the Twinkie Defense, and the legalome in forensic psychology, the question is how might the *absence* of these nutrients (omega-3 fatty acids, polyphenols, and vitamins/minerals) in an ultra-processed food-heavy diet [57,224,225], and the *presence* of mood-altering additives (e.g., glutamate flavor enhancers) [183,226] influence diminished capacity and intent? How does the intake of one nutrient (e.g., vitamin D) offset the potential adverse cognitive–behavioral consequences of flavor enhancers and other synthetic food additives [227]? How do these potentially harmful ingredients add to the addictive properties of ultra-processed foods [228], and how does the hyper-palatability [229] influence a vulnerable person toward ‘comfort food’ as a means to palliate isolation and depression, as claimed by the defense in *The People v White*. There is also a need to better understand the frequency of auto-brewery syndrome and how ultra-processed foods and dysbiosis might combine to produce alcohol and a host of other chemicals with forensic implications [18]. How could advances in breath tests (e.g., those that measure fasting and post-prandial metabolites) help researchers with better diagnostics that might be used in risk assessments? 

The subject of dietary tryptophan (as a precursor of serotonin) is also of importance to the relationship between ultra-processed foods and antisocial behavior. Preclinical studies show that high fat/high sugar diets can alter serotonergic gene expression, likely mediated by the microbiome [230]. Recent human studies have linked tryptophan-rich diets with better mental health and social cognition [231,232]. Dietary tryptophan appears to enhance emotion recognition, affiliation with victims, and cognitive empathy [233]. In addition, multiple experimental studies have linked dietary tryptophan depletion with increased aggression, quarrelsome behavior, and diminished agreeableness [234]. Ultra-processed foods in the Nova 4 category are typically not rich in tryptophan [235]. Given the widespread use of ultra-processed foods in carceral systems, how might the tryptophan content of the diet relate to outcomes ranging from rule infractions to acts of major violence in prisons? How might gut microbes, which produce various tryptophan metabolites, including those with neuroactive properties, factor into the links between dietary tryptophan and behavior [236]? 

Finally, the defense in *The People v. White* referred to the phenomenon of hypoglycemia as a possible explanation for violence. At the time of trial, the notion that low blood sugar, following the consumption of high-sugar, low-protein meals, is a contributor to neuropsychiatric complaints and aggressive behavior was supported by limited evidence [237,238]. In the years immediately after the trial, small-scale laboratory studies linked low blood glucose with aggressive reactions (measured after an oral glucose beverage), in both women and men [239,240]. Importantly, these associations were not dependent upon very low levels of blood glucose that typify diagnostic hypoglycemia. Researchers also reported that among criminal offenders (vs. healthy non-offender controls), an oral glucose challenge leads to initially higher levels of blood glucose and a subsequent lower glucose nadir in the period following glucose consumption [241,242,243,244]. More recent evidence supports the idea that, after initial consumption, glucose reduces aggressive tendencies and increases helping behavior under stress [245,246]. However, this benefit may be short-lived. Low blood glucose is associated with aggression in healthy adults [207,247]. Is it possible that a vulnerable person might resort to aggression in the period following the consumption of a high-sugar ultra-processed food, as blood glucose levels fall? Initial observations linking violence with both low blood glucose (after glucose challenge) and deficits in central neurotransmission [248] are worthy of further investigation. 

## 9. Conclusions

Although the roots of neurolaw and the use of objective measurements (e.g., EEG) in the courts date back almost a century, recent years have witnessed an increased uptake of neuroscience findings in criminal and civil courts. Discoveries in the neurosciences and related interdisciplinary fields, especially those that illuminate structure/function relationships to behavior, are actively challenging traditional legal assumptions surrounding blame, responsibility, criminal intent, agency, and moral responsibility [249]. While neurolaw does not challenge society’s right to protect itself, and to separate individuals that present a danger, it does confront matters of punishment and notions of equally distributed free will [250]. Among the exciting advances in neuroscience-related research, the role of nutrition and synthetic additives in nervous system structure and function has received increased attention. In addition, microbiome studies, especially preclinical work involving fecal transplants, are indicating that gut microbes make a significant contribution to mammalian cognition and behavior. Since the microbiome is dependent upon diet and other environmental factors, it is no longer possible to dismiss nutrition as an important factor in neurocognition [251]. 

In forensic neuropsychology texts and the popular press, the Twinkie Defense is often claimed to be a ‘myth’. However, using court transcripts we have shown that discussions of ultra-processed foods as a causative factor were suggested in testimony and in closing arguments, and attempts were made by the prosecution to refute causal connections. The case had a chilling effect on the defense of diminished capacity, depreciated trust in neuropsychiatric testimony, and likely minimized the seriousness of diet–brain–behavior interactions [252]. Still, even in the 1980s, the Twinkie Defense was credited with accelerating robust debate on the role of neuroscience in matters of criminal responsibility and the morality of punishing those with neuropsychiatric disorders [1]. Forty-five years later, emerging research in neurolaw and the study of the biological underpinnings of violence, aggression, antisocial behavior, and criminal behavior writ-large, underscore the terms intersectionality and vulnerability [14]. Based on genetics, epigenetics, lived experiences (and all of the associated adverse exposures or positive assets), and innumerable inputs across the spectrum of biopsychosocial factors (especially those related to antisocial behavior and justice involvement [253]), how might dietary inputs intersect with the vulnerabilities of an individual [254]? Based on the available evidence in the realm of nutritional criminology, we suspect that dietary inputs contribute to (and press upon existing) vulnerabilities of criminal behavior in ways that are more consequential than currently appreciated in the criminal justice system [26]. 

The American Psychological Association’s (APA) recent inclusion of Continuing Education (CE) modules on nutrition and behavior in its flagship *Monitor on Psychology*, has sent a signal that the topic of nutrition and behavior is here to stay. It is noteworthy that the lead article introducing the APA CE series acknowledged that researchers in nutritional neuroscience have historically “encountered skepticism and dismissiveness from many health care professionals and researchers, including psychologists, who tend to downplay the importance of diet in health and mental health” [255]. Based on increasingly robust mechanistic evidence, that dismissiveness is changing. Although the APA CE did not venture into the criminal justice system applications, the pace of nutritional neuroscience and attention to neurolaw, indicates that such applications are an inevitability. Although many unanswered questions remain, if the research continues to strengthen on its current trajectory, experts in forensic neurosciences can expect to be called upon with increased frequency, especially at the intersection of nutrition and diminished capacity. In the meantime, it is essential that critically relevant findings in neuroscience permeate criminal justice discourse.

## Data Availability

No new data were created or analyzed in this study. Data sharing is not applicable to this article.
